# Electrophysiology Models for End-Stage Renal Disease Maladaptations That Promote Asystole

**DOI:** 10.31083/RCM37400

**Published:** 2025-07-30

**Authors:** Ikeotunye Royal Chinyere

**Affiliations:** ^1^Sarver Heart Center, University of Arizona, Tucson, AZ 85724, USA; ^2^Division of Cardiology, Banner – University Medical Center, Tucson, AZ 85724, USA

**Keywords:** chronic kidney disease, arrhythmia, repolarization, cardiac, dialysis

## Abstract

Many patients with chronic renal impairment experience cardiac comorbidities throughout their lives, and the incidence of electrophysiological demise for patients with terminal renal impairment requiring renal replacement therapy is higher than in patients with normal renal function. Thus, this relationship warrants continued examination, such that the risk of subsequent cardiac complications might eventually be mitigated. This review aims to outline the electrophysiology concepts, both basic and clinical, underlying the pathophysiology mediated by end-stage renal disease (ESRD). An evaluation of how chronic kidney disease may accelerate adverse cardiac remodeling, as well as the mechanisms through which hemodialysis may precipitate electrophysiological aberrations that impair the ability of the conduction system to maintain normal sinus rhythm, are provided. Furthermore, relevant animal models for this pathophysiology, with respect to their innate ability to recapitulate human renal and cardiac electrophysiology, are outlined. Specifically, the concepts of hyperkalemia, pericarditis, and arrhythmia are discussed in relation to ESRD. Furthermore, murine, porcine, and human species are compared and contrasted on all structural levels, from subcellular to clinical, illustrating which models best recapitulate this propensity to asystole.

## 1. Introduction

End-stage renal disease (ESRD) is a laboratory diagnosis (estimated glomerular filtration rate less than 15 milliliters/minute/1.73 meters^2^) that also requires the presence of clinical signs and symptoms of uremia, such as anorexia, nausea, and fatigue. ESRD is the right-most position on the spectrum of acute and chronic renal syndromes that has an age-specific down-trending prevalence, which is offset by an increasing total prevalence in the setting of epidemic hypertension and diabetes [1].

Preceded by the progressive loss of nephrons, termed renal insufficiency, as well as functional impairment of blood filtration, termed renal failure [2], ESRD is a terminal condition that can be managed via dialysis, preferably peritoneal dialysis for young patients without other comorbidities [3], or renal transplantation. However, dialysis has adverse effects on the cardiovascular system through oxidative stress and on the metabolic system through hyperlipidemia and hyperhomocysteinemia [4]. A therapy superior to dialysis has yet to be revealed, with the exception of allogeneic renal transplantation, which necessitates lifelong immune modulation.

**Fig. 1.**
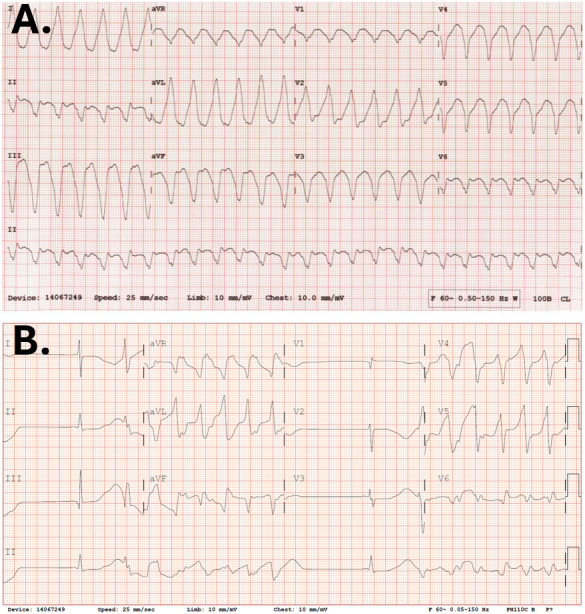
**Patient case**. (A) A surface electrocardiogram from a patient with end-stage renal disease on intermittent hemodialysis with comorbid multivessel coronary artery disease complicated by coronary artery bypass grafting and heart failure with reduced ejection fraction secondary to ischemic cardiomyopathy, who presented to the hospital via emergency medical services immediately after scheduled outpatient hemodialysis, where the patient experienced unwitnessed cardiac arrest secondary to sustained monomorphic ventricular tachycardia. (B) A follow-up surface electrocardiogram from the same patient, which illustrates additional reentrant ventricular ectopy in the setting of prolonged corrected QT interval despite normal serum electrolytes outside of iatrogenic mild hypermagnesemia.

The most common cause of death related to ESRD is not renal in nature but cardiovascular, often through atrial and ventricular tachyarrhythmias leading to acute asystole. Indeed, sudden cardiac death (SCD) represents 22% of ESRD-related deaths [5], with the arrhythmogenic substrate thought to be enhanced through the cardiovascular and metabolic remodeling associated with chronic kidney disease. Once sufficient proarrhythmic remodeling, which is mediated through both uremia and often comorbid subacute ischemia, has produced a critical volume of arrhythmogenic substrate with quenched repolarization reserve, it is hypothesized that the substrate is then activated by the sudden fluxes in electrolyte concentration, both relative and absolute, associated with hemodialysis (Fig. 1).

All of these concepts have remained active areas of research [6, 7, 8] as both physicians and molecular scientists pursue recapitulating the adverse cardiorenal remodeling in animal models to identify necessary and sufficient steps, ultimately aiming to improve clinical outcomes via novel interventions. However, some animal models are better suited to approximate human macroscopic renal and cardiovascular electrophysiology due to similarities in subcellular protein expression and comparable electromechanical coupling profiles.

## 2. End-Stage Renal Disease

ESRD is a dynamic disease with four major metabolic consequences: (1) hypertension due to blunted blood filtration for micturition, (2) components of uremic syndrome due to impaired elimination of nitrogenous waste, (3) components of metabolic acidosis due to hypertrophy-turned failure of residual nephrons causing retention of hydrogen ions, and (4) components of non-hemolytic normocytic anemia via low plasma erythropoietin, dialysis-mediated blood loss, and possibly inadequate nutritional intake of folate and cobalamin. While these four clinical findings are frequently encountered, this review will focus on the more subtle concepts of hyperkalemia, pericarditis, and arrhythmia to address the cardiac electrophysiology underlying ESRD-mediated cardiac arrest and SCD.

### 2.1 Hyperkalemia

Normal potassium homeostasis is predominantly a renal circadian process that occurs slowly, in contrast to the acute process that occurs over four hours or less with hemodialysis or less than one hour with emergent insulin, albuterol, and bicarbonate-mediated intracellular shifting. Hyperkalemia, often found during fasting periods in patients with ESRD, has been associated with low muscle tone, cardiac conduction deficits, and both bradycardic and tachycardic arrhythmias [4]. Moreover, the risk of all-cause mortality increases nearly exponentially with increasing serum potassium levels [9]. Given this exceptional degree of increased risk, it is hypothesized that the effects of excess extracellular potassium on the heart specifically are most related to the identified elevated all-cause mortality. Meanwhile, cellular adaptation to chronic hyperkalemia, characterized by aberrations in sodium, calcium, and chloride currents, may contribute to the background that underlies bradycardia, prolonged QT interval, and asystole during the peri-dialysis period [10, 11].

**Fig. 2.**
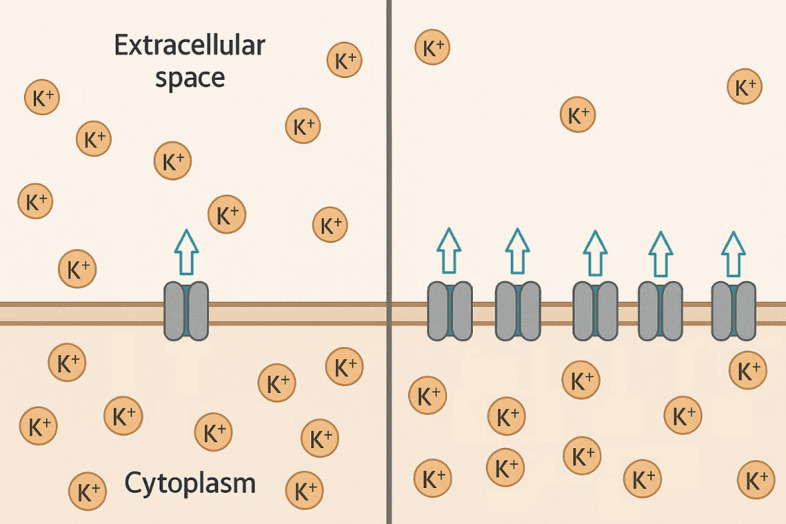
**Maladaptive potassium efflux regulation**. A stylized schematic of the non-uniform potassium efflux upregulation that could occur with recurrent hyperkalemia. This adaptation could become pathologic when serum potassium levels suddenly decrease from therapies, such as acute hemodialysis, creating repolarization heterogeneity and electrical dyssynchrony. Illustration modified from Microsoft Copilot May 2025 Update (1.25053.93.0).

**Fig. 3.**
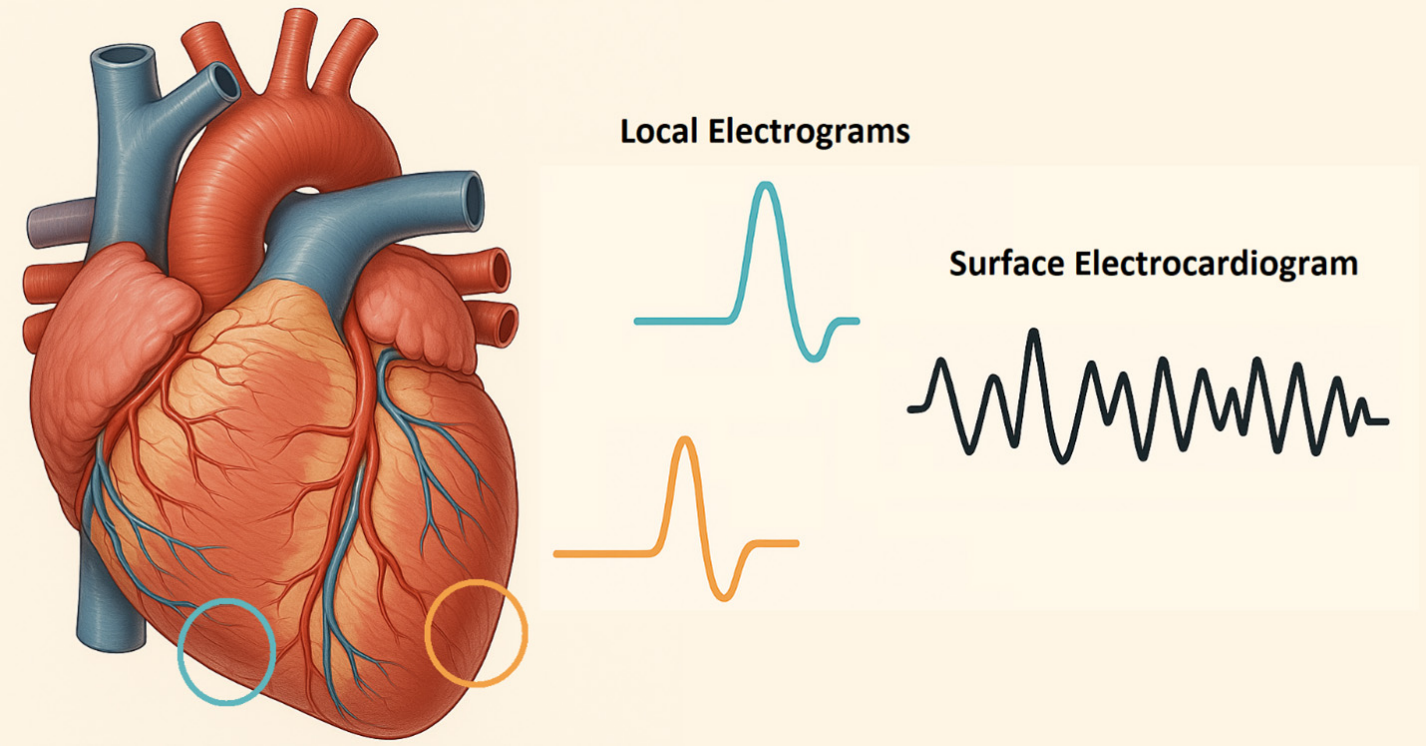
**Proarrhythmic repolarization heterogeneity**. An anatomic diagram of the geographically separated electrical dyssynchrony in the setting of rapid electrolyte changes. In combination with other secondary pathophysiology, end-stage renal disease and rapid hemodialysis can facilitate sudden cardiac death via ventricular tachyarrhythmia—illustration modified from Microsoft Copilot May 2025 Update (1.25053.93.0).

Additionally, it is hypothesized that the decreased driving force for potassium efflux in both autorhythmic and mechanically driven cardiomyocytes during chronic hyperkalemic periods leads to modulation of membrane channel abundance and distribution, in an attempt to maintain normal physiologic action during depolarization and repolarization. These non-uniform adaptive changes suddenly become maladaptive in the face of acute dialysis-mediated hypokalemia, where the driving force for potassium efflux is substantially increased (Fig. 2). Furthermore, this sudden increase in potassium efflux could lead to hyperpolarization and dispersed repolarization heterogeneity (Fig. 3). Thus, molecular studies are needed to evaluate this hypothesis, as potential therapeutic targets in the membrane channel expression pathway may be identified to interrupt this potential adaptive-turned maladaptive phenomenon, or even correct it acutely.

### 2.2 Pericarditis

Pericarditis in the context of ESRD is thought to be due to uremic toxin accumulation. The parietal and visceral pericardium are composed of mesothelial cells, fibroblasts, adipocytes, and small blood vessels. Uremic toxins, such as urea and the ensuing reactive oxygen species, parathyroid hormone, and homocysteine, can precipitate pericarditis by facilitating a proinflammatory state, particularly in adipose tissue [12]. Pericarditis is related to arrhythmias in that an inflammatory infiltrate from the visceral pericardium has been described extending to the sinus node and other portions of the atrial myocardium [13], which could explain why atrial fibrillation is prevalent in ESRD patients [14].

### 2.3 Arrhythmia

As previously demonstrated (Fig. 1), a disproportionate number of chronic dialysis patients consistently succumb to lethal ventricular arrhythmia. From the perspective of this review, multiple components are likely at play regarding the initiation and propagation of the ventricular arrhythmia. The first and most obvious factor is ESRD-mediated hyperkalemia, as well as other electrolyte imbalances that can contribute to creating an arrhythmogenic substrate. The second factor encompasses all uremic toxins that may impair normal myocardial function by affecting mitochondrial energetics [12] and induce macro- and microvascular atherosclerosis, leading to ischemia-mediated fibrosis. Note that this second factor is not a direct cause for arrhythmia, but rather for myocardial dysfunction that may, in itself, predispose to arrhythmia. The third and final factor, from the perspective of this review, involves pericarditis, as a non-compliant pericardium can serve as both a mechanical obstruction and a paracrine hindrance through its recruitment of proinflammatory cells and cytokines. Again, this third factor does not directly lead to arrhythmia, but rather contributes to the proarrhythmic environment.

## 3. Renal Ion Channels

Renal ion channel function modulates serum electrolyte values, which in turn can modulate cardiac ion channel density and function in a compensatory manner.

### 3.1 Humans

Renal tubule epithelium is one of the most active tissues in the entire body due to the diversity of channels and the sheer quantity of secondary active transport transcellular pumps. The sodium–potassium ATPase is at the core of all electrophysiology, predominantly represented by the α1–β1 isoform in mammalian kidneys [15], which establishes the baseline cation gradients that underpin ATP-independent electrophysiology in the body, accounting for the majority of all electrophysiologic activity. The basolateral ten + two transmembrane domain-containing enzyme secretes sodium and retains potassium; hence, why fluid overload-mediated hyponatremia, in addition to hyperkalemia, is a common finding in ESRD [16] since an osmotic gradient is created by the diminished tubular sodium–potassium ATPase activity, which leaves cations in the plasma. This reduced activity has three possible explanations: (1) uremic toxin-damaged mitochondrial machinery, stunting the basolateral sodium–potassium ATPase, (2) a defect in the apical potassium leak channels upon which the sodium–potassium–chloride symporter, namely NKCC2 isoforms A, B, and F [17, 18] rely, or (3) frank sloughing of the tubular epithelium.

The renal outer medullary potassium channel, specifically isoforms 1 and 3 of the principal cell [19], facilitates the efflux of potassium out of the cytoplasm and into the ultrafiltrate under the influence of magnesium [20] and in collaboration with the aldosterone-sensitive epithelial sodium channel. The large potassium channel also works in conjunction with the renal outer medullary potassium channel, with the exception that its lower affinity for potassium makes it flow-dependent in ultrafiltrate [19, 21, 22, 23]. In ESRD after chronic kidney disease, it is hypothesized that chronic perturbations in potassium concentrations induce an upregulation in excretion mechanisms (sodium–potassium ATPase and apical potassium channels) as well as altered handling of other electrolytes (sodium–potassium–chloride symporter, aldosterone-mediated epithelial sodium channel [24], and Wnk-sensitive sodium–chloride cotransporter [25]) to preserve membrane potentials at the expense of chemical gradients [26].

### 3.2 Mice

Murine nephropathy models are widely employed; however, their clinical application in relation to ESRD is limited. Moreover, the advantages conveyed in these models are often unrelated to their innate ability to recapitulate human physiology-turned-pathophysiology, but rather relate to logistical advantages such as decreased housing costs, feed costs, and ease of genetic and surgical experimentation.

It is no surprise that mouse renal function differs from that of humans. For starters, proximal tubular cells occupy a larger surface area than just the proximal tubule. Meanwhile, tubularization of Bowman’s capsule is commonly observed in our quadrupedal counterparts [27], while it is rare in humans. This could partially explain why a five-sixths nephrectomy induces an acutely elevated blood urea nitrogen and creatinine that then levels off to mild levels after four weeks [28, 29] in the commonly used C57BL/6 strain.

The other explanation for enhanced renal activity even in the face of pathology could be that mouse tubular epithelial cells are capable of withstanding a remarkable amount of oxidative stress [30], which may be more than human cells can combat. Nonetheless, both mice and humans are thought to express the same sodium–potassium ATPase α1–β1 isoforms, although with a different γ subunit [15, 31], which may partially explain the altered stability. While isoforms 1 and 3 of the renal outer medullary potassium channel are relevant in humans, isoform 1 has no functional significance in mice and is not coupled with the action of the sodium–potassium–chloride symporter as it is in humans [32].

### 3.3 Pigs

Swine models of nephropathy are considered superior to murine models due to the increased similarity in enzyme and transporter isoforms at the kidney level [33], as well as in other relevant organs, such as the heart, thereby enhancing overall physiologic translation ability. Notably, the degree of metabolic acidemia may play a significant role in determining the extent of nephrotoxicity and the subsequent translational utility between swine and mouse models [34]. The scientific literature contains a paucity of original research manuscripts evaluating acid–base homeostasis in mice with ESRD. Pigs that undergo four/five-sixths nephrectomy also exhibit an acute increase in BUN and creatinine that recovers to a moderate (meaning more often statistically significant) level [35], rather than the mild level of uremia observed in most mouse models (Table 1) [36].

**Table 1.** **Summary of renal differences**.

Category	Human	Mouse	Swine
Nephron count	∼1 million per kidney	∼14,000 per kidney	Intermediate between mice and humans
Na^+^/K^+^-ATPase	α1–β1 isoforms; essential for electrochemical gradients; impaired in ESRD	Similar isoforms but with a different γ subunit; altered stability	Similar to humans; high translational relevance
Potassium channels	ROMK isoforms 1 and 3 functional; BK channel flow-dependent	ROMK isoform 1 not functional; varied BK channel function	Similar structure and function to humans
Sodium transporters	Tightly-regulated NKCC2, ENaC, and NCC; hormone-responsive	Functional but less coupling; regulatory differences	Closely resemble human transporter expression and regulation
Water reabsorption	AQP2 regulated by vasopressin; essential for water reabsorption	Functional AQP2; possible differences in vasopressin response	Similar vasopressin response and function to humans
Response to injury	Chronic electrolyte imbalance in ESRD; impaired pumps and epithelial damage	Acute BUN/creatinine increase post-nephrectomy; mild uremia; stress-resilient	Moderate uremia post-nephrectomy; more closely mimics human ESRD

A table of the major differences between human, murine, and swine models with respect to renal function, ion channels, and renal disease severity. Definitions: α, alpha; β, beta; ESRD, end-stage renal disease; ROMK, renal outer medullary potassium channel; BK, big potassium/maxi-K; NKCC2, Na–K–2Cl cotransporter 2; ENaC, epithelial sodium channel; NCC, sodium–chloride cotransporter; AQP2, aquaporin 2; BUN, blood–urea–nitrogen.

Morphometry of the porcine kidney yields smaller dimensions than the human kidney in all parameters except for length [37]. However, their embryological development from pronephros to mesonephros to metanephros is identical to that of human and mouse renal development, as all are mammalian. The difference is attributed to the absolute number of nephrons per kidney, which is approximately 14,000 in mice, 1 million in humans, and a value interpolated for swine, although the number is highly variable between human ethnicities [38]. Swine arterial segments do not resemble those of humans with respect to distribution and size [39], while mice are thought to more consistently replicate the typical cranial–caudal organization [40, 41].

## 4. Pericarditis

As mentioned before, pericarditis in itself does not directly lead to arrhythmia, but rather is secondary to a proinflammatory trigger that may contribute to a proarrhythmic environment.

### 4.1 Humans

Pericarditis is an active process that consists of stages of inflammation, but can also refer to the residual sequelae from an acute bout of pericarditis. Frequently caused by viral infection or autoimmune hypersensitivity in middle-aged men, pericarditis is a rare diagnosis and elusive in that the exact mechanisms have yet to be defined. Clinically, the majority of cases of acute pericarditis are benign, perhaps lessening the driving force behind basic and translational research. With an estimated annual incidence of less than 1% among all hospitalized patients [42], the primary therapy for pericarditis remains non-steroidal anti-inflammatory drugs, with colchicine and interleukin-1 blockade treatments used in the event of recurrence.

The mechanism of pericarditis depends on which specific etiology is being discussed. As previously alluded to, the infectious etiology is commonly encountered; however, the uremic etiology may be more frequently represented in the scientific literature. Regardless of the specific triggers, pericarditis eventually involves activated resident fibroblasts, which have a high metabolic and telomere-conferred reproductive tolerance. This hyperplasia, preceded by the inflammatory secretome from both polymorphonuclear myeloid cells and lymphocytic cells, results in granulation tissue [43], which facilitates angiogenesis as the M1–M2 transition is completed [44, 45]. This inflammation may lead to myocarditis, and the increased mechanical resistance of the remodeled pericardium may hinder the ability of the right heart to function properly due to its relatively low muscle mass.

### 4.2 Mice

A review of the scientific literature reveals a dearth of research on murine pericarditis, as our search yielded only four manuscripts representing the years 1979 to 2018. With this limited data, one can draw the following conclusion: mice are susceptible to Coxsackievirus- and *Trypansoma cruzi-*induced myopericarditis [46, 47], likely due to either the activation of the Nod-like receptor type three protein innate immunity cascade [48] or activation of innate lymphoid cells [49].

Although limited, this conclusion supports the notion that murine models of human ESRD-mediated pericarditis may serve a purpose due to their analogous intrinsic qualities in immunology and pericardial physiology.

### 4.3 Pigs

While four manuscripts could be identified with murine pericarditis, three of which were contemporary, only two manuscripts could be identified that describe the modelling of pericarditis in porcine models, and only one of the two manuscripts utilized a clinically relevant methodology [50]. While the publication addresses the autoimmune etiology of pericarditis, it sheds little light on the uremic processes. It is hoped that this review will inspire further research in this area.

## 5. Ion Channels in the Heart

### 5.1 Humans

Human cardiomyocytes express α1, α2, and α3, in addition to β1 isoforms, to formulate their sodium–potassium ATPase [51]. The α2 isoform is fundamental to both cardiac and smooth muscle due to its role in calcium homeostasis [52] and is the target of cardiac glycosides [53]. In comparison to the nephron, the cardiomyocyte is less complex in terms of diversity and the number of enzymes and channels in its sarcolemma. However, the present channels combine to function seamlessly on the order of microseconds and produce a very interesting pathology when uncoupled.

The sodium–potassium ATPase is coupled with a sodium–calcium antiporter, commonly isoform 1, as intracellular calcium fluxes and the basal sarcoplasmic concentration must be tightly controlled to prevent altered genetic regulation for anabolic, catabolic, or apoptotic processes [54]. Ryanodine receptor 2 is the largest known ion channel and is also the isoform present in humans to release calcium from the sarcoplasmic reticulum [55]. The functionally coupled dihydropyridine receptor for the L-type calcium current is composed of five subunits, of which the α1 subunit is paramount in determining pore size and drug interactions [56]. In the human cardiomyocyte, α1 isoforms 1.2 and 1.3 are present, conferring a relatively high sensitivity to compounds such as nifedipine.

The incessant metabolic activity of the heart requires a high density of mitochondria to power the preferentially aerobic cellular machinery. In healthy states, the organ operates similarly to a well-oiled machine. However, in the ESRD states characterized by low cellular energy due to hyperlipidemia, toxemia resulting from urea-mediated reactive oxygen species, and aberrant calcium homeostasis caused by elevated parathyroid hormone, alongside a high density of mitochondria, can be crippling due to their ability to induce apoptosis and subsequent heart failure. The coupling of L-type calcium channels to mitochondria is a critical link in calcium regulation [57] and may help explain the beneficial effects of nifedipine therapy in chronic kidney disease and ESRD [58].

Autorhythmic cardiomyocytes should also be acknowledged, as their functional decline may contribute to the frequently encountered uremic bradycardia and sudden cardiac death [59]. In addition, their failing strength may decrease the likelihood of spontaneous return to normal sinus rhythm during dyskalemic-induced tachycardic episodes. Indeed, alterations in the Na_V_1.5 current have been shown to increase the likelihood of arrhythmia, potentially to a greater degree than modulated activity in other potassium currents [60, 61].

Hyperpolarization-activated cyclic nucleotide-modulated channel isoforms 1–4, responsible for the funny current, are primarily expressed in conduction system cells; however, structural cardiomyocytes can also express these channels during adverse remodeling [62]. Essentially, any deviation from normal ion channel number or distribution will disturb the funny current in autorhythmic cells and subsequently increase the likelihood of arrhythmia or decrease the likelihood of successful cardioversion to sinus rhythm. Computational models have implicated calcium in autorhythmic cell deterioration during ESRD-mediated bradycardia and SCD [63]. Furthermore, autorhythmic cells may exhibit a high tolerance to ischemia but a low tolerance to toxicity, and their adaptive response often involves a change in the number and distribution of ion channels [64, 65].

### 5.2 Mice

Some rodents have been described to lack the sodium–potassium ATPase α2 isoform, rendering cardiac glycosides relatively inert [15, 53]. However, mice express the α2 isoform in addition to the β2 isoform, both of which seem to be restricted to expression in the myocardium [52]. Moreover, the coupled sodium–calcium antiporter, isoforms 1 and 2, have been documented [66], suggesting that the basic cellular machinery for maintaining the quintessential cation gradients is similar to that described in humans.

Nonetheless, this notion of similar machinery does not persist in the discussion of calcium regulation, with the most obvious difference between mice and humans, with respect to cardiac function, being the large difference in resting heart rate, which is a macroscopic manifestation of the minute differences in protein isoform expression related to sodium current and intracellular calcium handling. Mice express the ryanodine receptor 2 [55] similarly to humans; however, the upstream Na_V_1.5 is composed of the α, β1, and β2 subunits [67], rather than the α subunit with different β subunits. It is the β subunits that confer thermodynamic stability to the pore-forming α subunit, modulating the open–inactive–closed cycle [68, 69].

Calcium reuptake from the sarcoplasm back into the sarco-endoplasmic reticulum is the responsibility of SERCA2a in all mammals, with a minor contribution from isoform 2b [70]. While subtleties may exist between species with respect to the calcium ATPase amino acid sequence, a larger degree of modulation is regulated via phospholamban, a negative allosteric modulator that is conserved between all mammals, and sarcolipin, another negative allosteric modulator that shares a binding site with phospholamban and is also likely conserved between all mammals [70]. The allosteric effects are mediated by the degree of protein abundance, which differs between atria (a lesser quantity) and ventricles (a greater quantity), as well as between different mammalian species, such as mice and humans.

Finally, autorhythmic cardiomyocytes in mice are equivalent to those in humans with the main difference being in the action potential duration, which is shorter in mice due to (1) a faster pacemaker potential from hyperpolarization-activated cyclic nucleotide-modulated channel isoforms 1–4 [60, 71, 72] and (2), axillary subunit isoform differences in t-type calcium channels. Functional decline is hypothesized to be causative of ESRD-mediated sinus bradycardia; however, no significant difference in heart rate was observed between wild-type and 5/6 nephrectomy mice, nor was there an increase in spontaneous or inducible arrhythmias (Table 2; Ref. [73]). These findings are attributed to the mechanism of kidney disease induction, the compensatory hypertrophy of the remaining 1/6 kidney that acts to compensate for the acute insult, a short period of chronic kidney disease-associated ion channel remodeling without sufficient time for systemic electrolyte concentration disturbances, and no functional deterioration to ESRD.

**Table 2.** **Murine heart rate and electrophysiologic evaluation**.

Sample	Heart Rate (beats per minute)	Induced VT	Non-Sustained VT (episodes)
Wildtype	421 ± 28	0/5 (0%)	1
Uremic	381 ± 59	0/2 (0%)	1

The intrinsic heart rate (beats per minute) and response to electrophysiologic evaluation (incidence of programmed electrical stimulation-induced ventricular tachycardia (VT)) in wildtype mice (n = 5) and 5/6 nephrectomy-mediated uremic mice (n = 2; four weeks of uremia). The definition of sustained VT in small animals is greater than fifteen consecutive premature ventricular contractions as defined in previous publications [73]. No statistically significant relationships exist between reported parameters. Heart rate is reported as mean ± standard error of the mean.

### 5.3 Pigs

Cardiovascular research has long utilized swine models due to their similarity to human cardiac anatomy and physiology, their propensity to spontaneous atherosclerosis, and their inability to form collateral anastomoses quickly. The scientific literature reveals no major differences between pigs and humans with respect to either ion channel isoforms or relative expression. Specifically, swine cardiomyocyte expression of the sodium–potassium ATPase [74], sodium–calcium antiporter [75, 76], calcium channels, and their regulators [77, 78] is similar to that in human cardiomyocytes in both normal and disease states (Table 3). However, there has been and continues to be a significant difference in the propensity of pigs to tachyarrhythmia and susceptibility to cardioversion after fibrillation [79].

**Table 3.** **Summary of cardiac differences**.

Category	Humans	Mice	Swine
Ion channel expression	Uremia may induce autorhythmic cell dysfunction, in addition to potentially inducing chronic adaptive changes in ion channel expression to preserve action potential integrity.	Similar to humans in basal sodium–potassium handling but with unique calcium sensitivity and cycling; overall, relatively refractory to uremia.	Less gap-junction distribution, otherwise similar to human expression in ion channel isoforms and relative abundance at baseline and in ESRD.
Potassium regulation	Transient outward potassium current plays a role in repolarization.	Prominent transient outward potassium current, similar to humans.	Minimal role of transient outward potassium current in repolarization, contributing to a relatively prolonged action potential duration.
Arrhythmia propensity	Moderate propensity to ventricular arrhythmia at baseline that increases in ESRD.	Low propensity to ventricular arrhythmia at baseline that does not change significantly with ESRD.	A higher propensity for ventricular tachyarrhythmia at baseline has not been well-characterized in ESRD.

The major differences between human, murine, and swine models with respect to ion channels that facilitate cardiac rhythm.

This difference could be rooted in a difference in potassium regulation on a subcellular level, specifically in the transient outward potassium current. This current is prominent in repolarization in humans and mice but plays a negligible role, if any role at all, in the action potential in pigs [80]. Further, the innate propensity of pigs to arrhythmia, namely atrial and ventricular premature complexes and fibrillation rather than the conduction blocks found in other models, has been quantified [81, 82], and was attributed to (1) a relatively prolonged action potential duration due to less potassium efflux, increasing the likelihood of R-on-T, and (2) a smaller gap-junction distribution [83, 84]. Though their propensity to arrhythmias makes swine difficult to manage during experimental studies, it also facilitates robust clinical translation when novel therapeutics, such as the inhibition of the sodium–hydrogen exchanger, are evaluated with positive outcomes [85].

## 6. Conclusion

This review discusses the topics of hyperkalemia, pericarditis, and arrhythmia in the context of animal models for ESRD. Additional research is necessary to validate the hypothesis that increased potassium efflux secondary to chronic hyperkalemia in ESRD directly predisposes to lethal ventricular arrhythmias. Furthermore, it is not currently known whether potassium dysregulation in the setting of rapidly shifting serum levels is sufficient and/or necessary to produce the clinical phenotype of arrhythmogenic asystole observed in ESRD patients. Translational research has been and will continue to be paramount to understanding and eventually preventing cardiac arrest in patients. Thus, understanding the molecular, biophysical, and subsequent macroscopic differences in ion handling and chronic adaptations between humans, pigs, and mice will enable increasingly precise arrhythmia research with accurate clinical correlation.
